# Omicron: A SARS‐CoV‐2 variant of real concern

**DOI:** 10.1111/all.15264

**Published:** 2022-02-28

**Authors:** Pia Gattinger, Inna Tulaeva, Kristina Borochova, Bernhard Kratzer, Doris Trapin, Anna Kropfmüller, Winfried F. Pickl, Rudolf Valenta

**Affiliations:** ^1^ Department of Pathophysiology and Allergy Research Division of Immunopathology Center for Pathophysiology, Infectiology and Immunology Medical University of Vienna Vienna Austria; ^2^ Laboratory for Immunopathology Department of Clinical Immunology and Allergy Sechenov First Moscow State Medical University Moscow Russia; ^3^ Institute of Immunology Center for Pathophysiology, Infectiology and Immunology Medical University of Vienna Vienna Austria; ^4^ Österreichische Gesundheitskasse Klinikum Peterhof Baden Austria; ^5^ Karl Landsteiner University of Health Sciences Krems Austria; ^6^ NRC Institute of Immunology FMBA of Russia Moscow Russia

**Keywords:** COVID‐19, Omicron, protective antibodies, SARS‐CoV‐2, vaccine, variants of concern

## CONFLICT OF INTEREST

Rudolf Valenta has received research grants from HVD Life‐Sciences, Vienna, Austria, WORG Pharmaceuticals, Hangzhou, China and from Viravaxx AG, Vienna, Austria. He serves as consultant for Viravaxx AG and WORG Pharmaceuticals. The other authors have no conflict of interest to declare.

## AUTHORS’ CONTRIBUTIONS

PG: Designed and performed experiments, analyzed data, wrote manuscript, and read manuscript; IT, KB, and AK: Performed experiments, analyzed data, read manuscript, and provided samples and clinical data; BK and DT: Performed experiments, analyzed data, and read manuscript; WFP: Analyzed data and read manuscript; RV: Analyzed data, wrote manuscript, read manuscript, and designed and supervized experiments.

To the Editor,

As of today (February 14, 2022), more than 410 million persons (https://coronavirus.jhu.edu/map.html) have reportedly been infected by SARS‐CoV‐2. Furthermore, mass production and global application of COVID‐19 vaccines have begun (Supplemental reference S3). Both factors certainly contribute to the fact, that although numbers of worldwide SARS‐CoV‐2 infections end of 2021 were more than double as high as in the end of 2020, the number of COVID‐19‐associated deaths has dropped to approximately 50% at the same time (https://coronavirus.jhu.edu/map.html). However, the immunity to SARS‐CoV‐2 which has been established so far is challenged by the appearance of SARS‐CoV‐2‐variants which may escape cellular (Supplemental reference S4) and antibody‐dependent immunity (Supplemental reference S5). The recently described variant of concern (VOC) Omicron, which has emerged in South Africa in November 2021, is spreading in the meantime rapidly all over the world and has become a matter of great concern because it shows more changes in the SARS‐CoV‐2 genome that may affect immunity as compared with earlier variants^1^ (Supplemental references S6–S9). In particular, Omicron has significantly more amino acid mutations in the SARS‐CoV‐2 receptor‐binding domain (RBD), which binds to the ACE2 receptor on human cells, as compared with previous SARS‐CoV‐2 variants^2^ (Table S1). Antibodies directed to RBD are critically important for virus‐neutralization because the RBD‐ACE2 interaction represents the port of entry for the virus into cells leading to its replication in the host and to the consecutive spreading in the population.^3^, ^4^ The ability of RBD‐specific antibodies to prevent RBD binding to ACE2 can be measured with surrogate molecular interaction assays,^5^ which mimic classical virus‐neutralization tests^3^ and can therefore be quickly adapted to newly emerging SARS‐CoV‐2 variants of concern by using RBDs from the corresponding virus variants.

**FIGURE 1:**
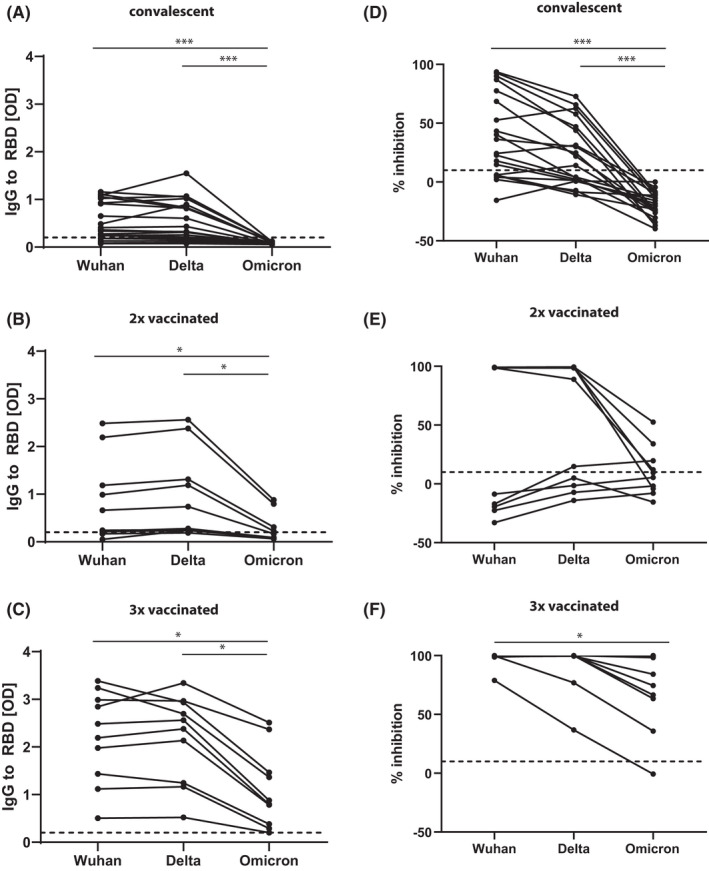
RBD‐specific IgG responses to variants of concern in convalescent patients and vaccinated subjects. Specific IgG reactivity (y‐axes: OD values correspond to bound IgG antibodies) in (A) COVID‐19 convalescent patients, (B) subjects vaccinated two times with licensed vaccines and (C) subjects vaccinated three times with licensed vaccines to RBD‐Wuhan, RBD‐Delta and RBD‐Omicron (x‐axes). Percentages of inhibition of binding (y‐axes) of RBD‐Wuhan, RBD‐delta and RBD‐Omicron (x‐axes) to ACE2 in (D) COVID‐19 convalescent patients, (E) subjects vaccinated two times with licensed vaccines and (F) subjects vaccinated three times with licensed vaccines. Dashed lines indicate cut‐offs. Significant differences between groups are indicated. *p* values: * <0.05, *** <0.001

**FIGURE 2:**
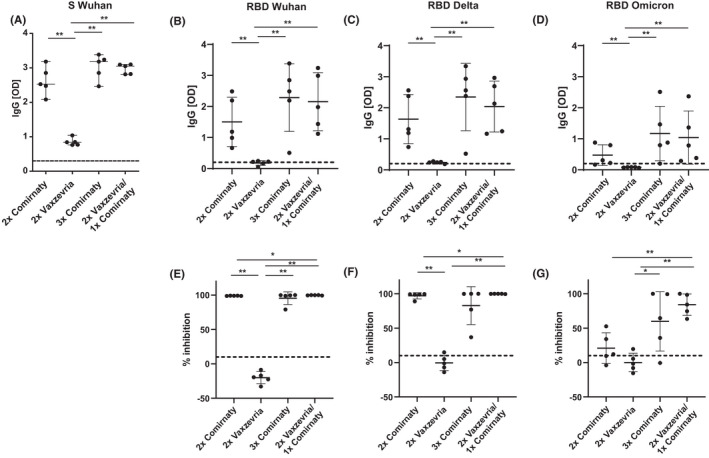
IgG response to RBD variants of concern after different vaccination strategies. Specific IgG reactivity (y‐axes: OD values correspond to bound IgG antibodies) in serum of subjects after different vaccination strategies (x‐axes) to (A) S protein‐Wuhan (B) RBD‐Wuhan, (C) RBD‐Delta and (D) RBD‐Omicron. Percentages of inhibition of binding (y‐axes) of (E) RBD‐Wuhan, (F) RBD‐Delta and (G) RBD‐Omicron to ACE2 by antibodies from subjects after different vaccination schemes (x‐axes). Dashed lines indicate cut‐offs. Significant differences between groups are indicated. *p* values: * <0.05, **<0.01, *** <0.001

Here, we compared the IgG recognition of RBD from the original Wuhan strain and recent variants of concern Delta (Pango B.1.617.2) and Omicron (Pango B.1.1.529) (Table S1) using sera from a random sample of adult COVID‐19 convalescent patients (Table S2: C1‐C20) and a random sample of adult subjects vaccinated two times (Table S3: D1‐D10) or three times (Table S3: T1‐T10) with a registered vector‐ (i.e., Vaxzevria) and/or mRNA‐based vaccine (i.e., Comirnaty) (Figures 1 and 2; Table S4). Furthermore, we studied the ability of antibodies in these sera to inhibit the binding of RBD‐Wuhan, RBD‐Delta, and RBD‐Omicron to ACE2 using the RBD‐ACE2 molecular interaction assay described by Gattinger et al.^5^ (Figures 1 and 2, Table S4). Sera from convalescent patients had been obtained from April to July 2020,^3^ 43–92 days (median 57.5 days) after the PCR confirmation of SARS‐CoV‐2 infection, sera from subjects vaccinated two times had been collected 26–31 days (median 27.5 days), and samples from subjects vaccinated three times were collected 23–40 days (median 28 days) after the last vaccination, respectively (Tables S2 and S3). There were no significant differences, regarding the levels of IgG antibodies specific for RBD‐Wuhan (Table S4: Median OD C1‐C20: 0.385; Median OD D1‐D10: 0.453; Median OD T1‐T10: 2.339) and RBD‐Delta (Table S4: Median OD C1‐C20: 0.379; Median OD D1‐D10: 0.509; Median OD T1‐T10: 2.470) (Table S4, Median reduction in binding comparing RBD‐Wuhan with RBD‐Delta: 4.3%), whereas RBD‐Omicron‐specific IgG levels (Table S4: Median OD C1‐C20: 0.073; Median OD D1‐D10: 0.128; Median OD T1‐T10: 0.836) were significantly lower than those specific for RBD‐Wuhan (Table S4, Median reduction of binding: 81.2%) and for RBD‐Delta in the convalescent patients and vaccinated subjects (Figure 1A‐C; Table S4).

The RBD‐specific IgG levels were in agreement with the results obtained regarding the inhibition of the RBD‐ACE2 interaction by serum antibodies (Figure 1D‐F). Antibodies from convalescent patients inhibited the binding of RBD‐Wuhan and of RBD‐Delta to ACE2 significantly stronger than the binding of RBD‐Omicron to ACE2 (Figure 1D). In fact, RBD‐Omicron binding to ACE2 was not inhibited by sera from convalescent patients in a relevant manner (Figure 1D). The inhibition of RBD‐Omicron binding to ACE2 by sera from subjects who had received two immunizations was much lower than that observed for RBD‐Wuhan (Table S4, Median reduction in inhibition 87.8%) and RBD‐Delta but did not reach significance because a considerable number of these subjects vaccinated with Vaxzevria mounted significantly lower levels of S‐ and RBD‐specific antibodies than those vaccinated twice with Comirnaty (Figure 2). Lower induction of Alpha and Delta neutralizing antibodies by two doses of Vaxzevria as compared with two doses of Comirnaty was also noted in another recent study.^6^ In subjects vaccinated three times, the inhibition of RBD‐Omicron binding to ACE2 was significantly lower than that of RBD‐Wuhan binding to ACE2 (Table S4, Median reduction of inhibition 27.7%) with two out of ten subjects (i.e., T1, T3, and Table S4) showing less than 50% inhibition.

Figure 2 shows that the IgG antibody levels specific for S, RBD‐Wuhan, RBD‐Delta, and RBD‐Omicron were higher in subjects who had received three immunizations than in those who had received two immunization, and this difference was significant for two doses of Vaxzevria. In fact, the inhibition of the binding of RBD‐Wuhan, RBD‐Delta, and RBD‐Omicron to ACE2 was higher in subjects immunized with two doses of Comirnaty than in those who had received two doses of Vaxzevria (Figure 2E‐G).

Median RBD‐Omicron‐specific IgG levels were lower in subjects having received 2 doses of Vaxzevria and a third dose of Comirnaty, than in those who had been immunized with three doses of Comirnaty but this difference was not significant (Figure 2D). Interestingly, the median inhibition of RBD‐Omicron binding to ACE2 was better for subjects treated 2xVaxzevria/1xComirnaty than for subjects treated with three doses Comirnaty (Figure 2G) but this difference was also not statistically significant.

To study if the degree of inhibition in the RBD‐ACE2 interaction of the variants is depending only on the levels of RBD‐specific antibodies or if also other factors such as specificity and/or avidity of antibodies^7^ may play a role, we analyzed RBD levels and percentages of inhibition in parallel (Table S4). We found, that certain subjects (e.g., T1 and T3, Table S4) had relatively low levels of RBD‐Omicron‐specific IgG, and accordingly, there was no (i.e., T1) or low (i.e., T3) inhibition of RBD‐Omicron binding to ACE2. However, we also found subjects with low levels of RBD‐Omicron‐specific IgG (i.e., T7, T8, and T10) with high inhibition of RBD‐Omicron binding to ACE 2 (Table S4). This result together with the finding that the RBD‐Omicron binding to ACE2 was even enhanced >20% for several convalescent patients (Figure 1D; Table S4: C1, C2, C4, C5, C9, C11, C16, C17, C19, and C20) would suggest, that factors, such as specificity and thus ability to form immune complexes^7^ as well as affinities/avidities of antibodies and not only their levels may guide the RBD‐Omicron‐ACE2 interaction.^5^, ^7^ In fact, we^5^ and later others^8^ noticed that sera from convalescent patients contain antibodies which seemed to be capable of forming immune complexes with RBD. Our current results indicate that this may also occur after vaccination. It is thus possible that such antibodies may form immune complexes with virus and/or S antigen produced after genetic vaccination at certain ratios of antibodies and antigen which then may result in antibody‐dependent enhancement (ADE) of disease or other side effects but this has not yet been demonstrated.

It may be considered as limitation of our study that we have only investigated antibody responses and their effects on the binding of RBD to ACE2 in molecular interaction assays and in a relatively limited number of subjects. However, our results are supported by three other very recent studies: One showed reduced neutralization of Omicron as compared with other variants after two doses of Vaxzevria or Comirnaty and in convalescent/vaccinated subjects^6^ and two others showing that even after three doses of Comirnaty neutralization of Omicron was lower than that of previous variants.^1^, ^9^ Our study provides additional information as it indicates that cross‐vaccination with two doses Vaxzevria followed by a booster with Comirnaty may eventually provide slightly better Omicron neutralization than three vaccinations with Comirnaty but further studies are needed to confirm this. In summary, we demonstrate that RBD‐Omicron is recognized much less by IgG antibodies from convalescent patients and by subjects immunized with vaccines based on SARS‐CoV‐2 Wuhan, even when immunized three times. Furthermore, antibodies from convalescent patients and vaccinated subjects inhibited the interaction of RBD‐Omicron to ACE2 much less than the interaction between RBD‐Wuhan and RBD‐Delta and ACE2, respectively. Omicron‐induced disease severity seems to be lower due to possible intrinsic features of this variant and/or the fact that a considerable proportion of the population has developed SARS‐CoV‐2‐specific T cell (Supplemental reference S10) and antibody responses. However, Omicron has re‐infected a large number of convalescent and vaccinated subjects which according to our results may be attributed to the reduced capacity of antibodies specific for earlier variants to inhibit the binding of Omicron to the ACE2 receptor. SARS‐CoV‐2‐protective antibody responses have been shown to drop relatively quickly, and Omicron has now shown that SARS‐CoV‐2 variants can develop which escape protective antibody responses specific for earlier variants induced by infection or vaccination. Therefore, Omicron appears to be a variant of real concern, especially for vulnerable persons, and it will be important to adapt vaccines and vaccination strategies to SARS‐CoV‐ 2 Omicron and newly evolving escape variants. This may be achieved by combination vaccines including the most divergent SARS‐CoV‐2 variants capable of inducing broad immunity.

## Supporting information

supinfo

Table S1

Table S2

Table S3

Table S4
