# Supplementary material for: Omicron: A SARS‐CoV‐2 variant of real concern
Source: Allergy. 2022 Feb 28;77(5):1616–20. doi: 10.1111/all.15264 (PMC9111213; doi:10.1111/all.15264)
Supplement: Supplementary file 1 — supinfo [file ALL-77-1616-s001.docx]

**Online Repository**

**Omicron: A SARS-CoV-2 variant of real concern**

Pia Gattinger, PhD^a^, Inna Tulaeva, PhD^a,b^, Kristina Borochova, PhD^a^, Bernhard Kratzer, PhD^c^, Doris Trapin, MSc^c^, Anna Kropfmüller, MD^d^, Winfried F. Pickl, MD^c,e^, Rudolf Valenta, MD^a,b,e,f *^

^a^Department of Pathophysiology and Allergy Research, Division of Immunopathology, Center for Pathophysiology, Infectiology and Immunology, Medical University of Vienna, Vienna, Austria

^b^Laboratory for Immunopathology, Department of Clinical Immunology and Allergy, Sechenov First Moscow State Medical University, Moscow, Russia.

^c^Institute of Immunology, Center for Pathophysiology, Infectiology and Immunology, Medical University of Vienna, Vienna, Austria

^d^Österreichische Gesundheitskasse, Klinikum Peterhof, Baden, Austria

^e^Karl Landsteiner University of Health Sciences, Krems, Austria.

^f^NRC Institute of Immunology FMBA of Russia, Moscow, Russia.

* Corresponding author

Rudolf Valenta

Department of Pathophysiology and Allergy Research

Medical University of Vienna

Waehringer Guertel 18-20

A-1090 Vienna, Austria

Tel: +43-1-40400-51080

Fax: +43-1-40400-51300

E-mail: rudolf.valenta@meduniwien.ac.at

**Table Legends**

**Table S1.** Sequence alignment of SARS-CoV-2 RBD-Wuhan with RBDs from variants of concern.

**Table S2.** Characterization of COVID-19 convalescent patients.

**Table S3.** Characterization of vaccinated subjects

**Table S4.** Overview of S- and RBD-specific IgG antibody levels and of inhibitions of the RBD-ACE2 interaction in convalescent patients and vaccinated subjects.

**Materials and Methods**

**Sera and ethics statement**

Sera from COVID-19 convalescent patients (group C: n=20) were obtained from April to July 2020, approximately 8 weeks after confirmation of a SARS-CoV-2 infection by PCR when the subjects had recovered completely^S1^ (Table S2).

Sera from subjects without evidence for a prior SARS-CoV-2 infection who had been vaccinated with licensed SARS-CoV-2 vaccines were obtained approximately 4 weeks after administration of the last dose of the vaccine (Table S3). A prior SARS-CoV-2 infection of the vaccinated subjects was excluded by careful medical anamnesis, by the fact that despite according to extensive testing for SARS-CoV-2 infections in Austria according to the 3G rule (Getestet: tested; Geimpft: vaccinated; Genesen: convalescent) these subjects never had a positive PCR and/or antigen test results and most importantly they lacked antibodies specific for the nucleocapsid antigen which is highly indicative of a prior SARS-CoV-2 infection and not contained in any of the vaccines which the subjects had received. Ten subjects were vaccinated two times (group D) and 10 subjects were vaccinated three times (group T) according to the vaccination regimen recommended by of the Austrian health authorities in accordance with the European Medicines Agency (EMA) follows:

Subjects D1-D5 and T1-T5 (Table S3) were vaccinated with Comirnaty (BioNTech, Mainz, Germany) in a 5-week interval. Subjects D6-D10 and T6-T10 (Table S3) were vaccinated with Vaxzevria (AstraZeneca, Cambridge, UK) in a 12-week interval. Subjects T1-T10 (S3) had the booster vaccination with Comirnaty (BioNTech) 6 months after the second dose.

The study was performed according to the Declaration of Helsinki and approved by the Ethics committee of the Medical University of Vienna, Austria (EK 1302/2020). Signed informed consent was obtained from the study subjects.

**ELISA and molecular interaction assay**

Specific IgG levels to SARS-CoV-2 S protein and RBD as well as RBD from variants of concern (VOC) Delta and Omicron were measured as previously described^S1,S2^. In brief, S protein Wuhan (Genscript, Leiden, Netherlands), RBD-Wuhan (Genscript), RBD-Delta (Sino Biological, Beijing, P.R.China.) and RBD-Omicron (Sino Biological) were coated at 2 µg/ml in PBS overnight onto NUNC Maxisorb 96 well plates (Thermofisher, Thermo-Fisher Scientific, Waltham, MA, USA). Plates were blocked three hours with 3% BSA/PBST and subsequently incubated with serum samples (1:100 diluted) overnight at 4°C. Bound human IgG antibodies were detected by incubation with 1:1000 diluted HRP-conjugated anti-human IgG (BD, San Jose, CA, USA) for 2 hours and subsequent staining with ABTS (Sigma-Aldrich, St. Louis, MO, USA). Optical density was measured at 405/492 nm with an Infinite F50 ELISA reader (Tecan, Männedorf, Switzerland). A cut-off at OD=0.2 was determined with historic control sera as described^5^.

Calculations of percentages of reduction in IgG binding to RBD Wuhan versus RBD Delta and versus RBD Omicron (Table S4) were performed as follows: % reduction = 100-(100x OD_RBD-Delta or RBD-Omicron_/OD_RBD-Wuhan_). When no decrease in OD values corresponding to IgG binding to RBD Wuhan versus IgG binding to RBD Delta or Omicron was observed the % reduction was set to 0.

To measure the inhibition of RBD to ACE2 receptor binding by patients` sera, the molecular interaction assay was performed as described^S1,S2^.

In brief, 1:2 diluted serum was incubated for 3 hours with 50 ng of His-tagged RBD-Wuhan (Genscript), RBD-Delta (Sino Biological) and RBD-Omicron (Sino Biological) followed by a 3-hours overlay onto plate-bound ACE2 (2 µg/ml) (Genscript). Bound RBD was detected with a mouse monoclonal anti-His antibody followed by a HRP-labelled anti-mouse IgG_1_ antibody. Detection with ABTS as substrate and measurement of the colour reactions was done as described above. The percentages of inhibition were calculated as described^S2^. Cut-off at 10% inhibition was determined with historic control sera^7^.

The calculation of % reduction in inhibition (Table S4) when comparing the inhibitions obtained with sera for RBD Wuhan versus RBD Delta or RBD Omicron was performed as follows: Values <10% were set to 0. Percentage reduction = 100-(100x %Inhibition_RBD-Delta or RBD-Omicron_/%Inhibition _RBD-Wuhan_).

All determinations were performed in duplicates and each result is an average of duplicate determinations with <5% difference between the individual values.

**Statistics**

No statistical methods were used to predetermine sample sizes. All analyses were performed using GraphPad Prism Version 5.00 (La Jolla, CA, USA). Differences in immunoglobulin reactivity to proteins or differences of inhibition of receptor binding were determined using non-parametric two-tailed Mann-Whitney *U*-test. p values of <0.05 were considered as significant.

**Supplemental references**

S1. Gattinger P, Niespodziana K, Stiasny K, et al. Neutralization of SARS-CoV-2 requires antibodies against conformational receptor-binding domain epitopes. *Allergy*. 2022;77(1):230-242. doi:10.1111/all.15066

S2. Gattinger P, Borochova K, Dorofeeva Y, et al. Antibodies in serum of convalescent patients following mild COVID-19 do not always prevent virus-receptor binding. *Allergy*. 2021;76(3):878-883. doi:10.1111/all.14523

S3. Sampath V, Rabinowitz G, Shah M, et al. Vaccines and allergic reactions: The past, the current COVID-19 pandemic, and future perspectives. *Allergy*. 2021;76(6):1640-1660. doi:10.1111/all.14840

S4. Agerer B, Koblischke M, Gudipati V, et al. SARS-CoV-2 mutations in MHC-I-restricted epitopes evade CD8^+^ T cell responses. *Sci Immunol*. 2021;6(57):eabg6461. doi:10.1126/sciimmunol.abg6461

S5. Chen RE, Zhang X, Case JB, et al. Resistance of SARS-CoV-2 variants to neutralization by monoclonal and serum-derived polyclonal antibodies. *Nat Med*. 2021;27(4):717-726. doi:10.1038/s41591-021-01294-w

S6. Cao Y, Wang J, Jian F, et al. Omicron escapes the majority of existing SARS-CoV-2 neutralizing antibodies. *Nature.* 2021 Dec 23. doi: 10.1038/s41586-021-04385-3. Online ahead of print. PMID: 35016194

S7 Lobiuc A, Șterbuleac D, Sturdza O, et al. A conservative replacement in the transmembrane domain of SARS-CoV-2 ORF7a as a putative risk factor in COVID-19. *Biology* (Basel). 2021 Dec 5;10(12):1276. doi: 10.3390/biology10121276. PMID: 34943191

S8 Hachim A, Kavian N, Cohen CA, et al. ORF8 and ORF3b antibodies are accurate serological markers of early and late SARS-CoV-2 infection. *Nat Immunol.* 2020 Oct;21(10):1293-1301. doi: 10.1038/s41590-020-0773-7. Epub 2020 Aug 17. PMID: 32807944

S9 Weingarten-Gabbay S, Klaeger S, Sarkizova S, et al. Profiling SARS-CoV-2 HLA-I peptidome reveals T cell epitopes from out-of-frame ORFs. *Cell.* 2021 Jul 22;184(15):3962-3980.e17. doi: 10.1016/j.cell.2021.05.046. Epub 2021 Jun 3. PMID: 34171305

S10 Keeton R, Tincho MB, Ngomti A, et al. T cell responses to SARS-CoV-2 spike cross-recognize Omicron. *Nature.* 2022 Jan 31. doi: 10.1038/s41586-022-04460-3. Online ahead of print. PMID: 35102311
