# Supplementary material for: Omicron: A SARS‐CoV‐2 variant of real concern
Source: Allergy. 2022 Feb 28;77(5):1616–20. doi: 10.1111/all.15264 (PMC9111213; doi:10.1111/all.15264)
Supplement: Supplementary file 2 — Table S1 [file ALL-77-1616-s003.docx]

**Table S1.** Sequence alignment of SARS-CoV-2 RBD (Wuhan, Genbank accession Nr.: QHD43416.1) with the amino acid sequences from RBD variants^1^. Different amino acids are indicated, identical amino acids are indicated by dots and positions in the S protein are given on the margins. The sequences were colored to illustrate features of the amino acids (light red = acidic hydrophilic, yellow= neutral, light green=basic hydrophilic, light blue=hydrophobic).

Wuhan 330 PNITNLCPFGEVFNATRFASVYAWNRKRISNCVADYSVLYNSASFSTFKCYGVSPTKLND 390

Delta 330 ............................................................ 390

Omcrn 330 .........D...............................L.P.F.............. 390

Wuhan 391 LCFTNVYADSFVIRGDEVRQIAPGQTGKIADYNYKLPDDFTGCVIAWNSNNLDSKVGGNY 450

Delta 391 ............................................................ 450

Omcrn 391 ...........................N......................K.....S... 450

Wuhan 451 NYLYRLFRKSNLKPFERDISTEIYQAGSTPCNGVEGFNCYFPLQSYGFQPTNGVGYQPYR 510

Delta 451 ..R.........................K............................... 510

Omcrn 451 ...........................NK.....A........R..S.R..Y...H.... 510

Wuhan 511 VVVLSFELLHAP 522

Delta 511 ............ 522

Omcrn 511 . ........... 522

^1^Sequence variants of Delta (Pango B.1.617.2) and Omicron (omcrn) (Pango B.1.1.529) are indicated. Designations follow the Pango nomenclature (<https://cov-lineages.org/>).
