# Supplementary material for: Omicron: A SARS‐CoV‐2 variant of real concern
Source: Allergy. 2022 Feb 28;77(5):1616–20. doi: 10.1111/all.15264 (PMC9111213; doi:10.1111/all.15264)
Supplement: Supplementary file 3 — Table S2 [file ALL-77-1616-s004.docx]

**Table S2.** Characterization of COVID-19 convalescent patients

| **ID** | **Gender^1^** | **Age** | **COVID-19 Symptoms** | **blood sampling [days after pos. PCR]** |
| --- | --- | --- | --- | --- |
| C1 | f | 43 | mild | 56 |
| C2 | f | 49 | mild | 50 |
| C3 | m | 57 | mild | 57 |
| C4 | m | 37 | mild | 48 |
| C5 | m | 51 | mild | 59 |
| C6 | f | 34 | severe | 43 |
| C7 | m | 33 | mild | 45 |
| C8 | f | 75 | mild | 61 |
| C9 | m | 76 | mild | 61 |
| C10 | m | 58 | mild | 56 |
| C11 | m | 48 | mild | 52 |
| C12 | m | 54 | mild | 58 |
| C13 | m | 48 | mild | 62 |
| C14 | m | 40 | severe | 56 |
| C15 | f | 57 | severe | 61 |
| C16 | f | 49 | severe | 75 |
| C17 | f | 46 | severe | 53 |
| C18 | f | 55 | severe | 76 |
| C19 | m | 47 | severe | 92 |
| C20 | m | 46 | severe | 68 |
| **Median**  (Range) |  | **48.5**  (33-76) |  | **57.5**  (43-92) |

^1^f = female, m = male
