# Supplementary material for: Omicron: A SARS‐CoV‐2 variant of real concern
Source: Allergy. 2022 Feb 28;77(5):1616–20. doi: 10.1111/all.15264 (PMC9111213; doi:10.1111/all.15264)
Supplement: Supplementary file 4 — Table S3 [file ALL-77-1616-s002.docx]

**Table S3.** Characterization of vaccinated subjects.

| **ID** | **Gender^1^** | **Age** | **1st Vaccine** | **2nd Vaccine** | **3rd Vaccine** | **blood sampling [days after last vaccination]** |
| --- | --- | --- | --- | --- | --- | --- |
| D1 | m | 30 | Comirnaty | Comirnaty | n.d | 27 |
| D2 | m | 55 | Comirnaty | Comirnaty | n.d | 27 |
| D3 | f | 33 | Comirnaty | Comirnaty | n.d | 31 |
| D4 | m | 39 | Comirnaty | Comirnaty | n.d | 27 |
| D5 | m | 31 | Comirnaty | Comirnaty | n.d | 26 |
| D6 | m | 34 | Vaxzevria | Vaxzevria | n.d | 27 |
| D7 | f | 60 | Vaxzevria | Vaxzevria | n.d | 29 |
| D8 | f | 50 | Vaxzevria | Vaxzevria | n.d | 28 |
| D9 | m | 56 | Vaxzevria | Vaxzevria | n.d | 28 |
| D10 | f | 62 | Vaxzevria | Vaxzevria | n.d | 28 |
| **Median**  (Range) |  | **44.5**  (30-62) |  |  |  | **27.5**  (26-31) |
| T1 | f | 67 | Comirnaty | Comirnaty | Comirnaty | 23 |
| T2 | f | 26 | Comirnaty | Comirnaty | Comirnaty | 26 |
| T3 | f | 34 | Comirnaty | Comirnaty | Comirnaty | 28 |
| T4 | f | 34 | Comirnaty | Comirnaty | Comirnaty | 28 |
| T5 | m | 38 | Comirnaty | Comirnaty | Comirnaty | 30 |
| T6 | f | 39 | Vaxzevria | Vaxzevria | Comirnaty | 28 |
| T7 | f | 62 | Vaxzevria | Vaxzevria | Comirnaty | 28 |
| T8 | m | 40 | Vaxzevria | Vaxzevria | Comirnaty | 25 |
| T9 | f | 26 | Vaxzevria | Vaxzevria | Comirnaty | 34 |
| T10 | f | 50 | Vaxzevria | Vaxzevria | Comirnaty | 40 |
| **Median**  (Range) |  | **38.5**  (26-67) |  |  |  | **28**  (23-40) |

^1^f = female, m = male
