# Supplementary material for: Omicron: A SARS‐CoV‐2 variant of real concern
Source: Allergy. 2022 Feb 28;77(5):1616–20. doi: 10.1111/all.15264 (PMC9111213; doi:10.1111/all.15264)
Supplement: Supplementary file 5 — Table S4 [file ALL-77-1616-s005.docx]

**Table S4.** Overview of S- and RBD-specific IgG antibody levels and of inhibitions of the RBD-ACE2 interaction in convalescent patients and vaccinated subjects.

| **ID** | **IgG to [OD]** | | | | **Reduction in binding [%]^1^** | | **% inhibition^2^** | | | **Reduction in inhibition [%]^3^** | |
| --- | --- | --- | --- | --- | --- | --- | --- | --- | --- | --- | --- |
|  | **S** | **RBD Wuhan** | **RBD Delta** | **RBD Omicron** | **Wuhan to Delta** | **Wuhan to Omicron** | **RBD Wuhan** | **RBD Delta** | **RBD Omicron** | **Wuhan to Delta** | **Wuhan to Omicron** |
| C1 | 2.312 | 0.911 | 0.819 | 0.115 | 10.2 | 87.4 | 40.3 | 3.6 | -30.4 | 100 | 100 |
| C2 | 2.207 | 1.057 | 0.812 | 0.123 | 23.1 | 88.4 | 68.6 | 22.0 | -24.5 | 67.9 | 100 |
| C3 | 0.404 | 0.076 | 0.075 | 0.055 | 1.6 | 28.1 | 14.8 | 0.9 | 0.1 | 100 | 100 |
| C4 | 0.411 | 0.182 | 0.163 | 0.066 | 10.4 | 64.1 | 5.5 | -10.6 | -23.3 | 0 | 0 |
| C5 | 0.981 | 0.361 | 0.314 | 0.069 | 13.1 | 80.8 | 18.0 | 2.4 | -20.4 | 100 | 100 |
| C6 | 1.043 | 0.244 | 0.255 | 0.074 | 0.0 | 69.7 | 4.1 | 1.4 | -18.3 | 0 | 0 |
| C7 | 0.808 | 0.182 | 0.195 | 0.064 | 0.0 | 65.0 | 22.8 | 4.1 | -13.7 | 81.8 | 100 |
| C8 | 1.113 | 0.339 | 0.325 | 0.064 | 3.9 | 81.2 | 36.4 | 30.0 | -9.6 | 17.5 | 100 |
| C9 | 1.945 | 0.653 | 0.604 | 0.074 | 7.4 | 88.6 | 43.4 | 24.8 | -34.1 | 42.9 | 100 |
| C10 | 0.321 | 0.087 | 0.096 | 0.062 | 0.0 | 28.7 | 5.7 | -8.6 | -11.5 | 0 | 0 |
| C11 | 2.276 | 1.107 | 0.832 | 0.114 | 24.9 | 89.7 | 90.1 | 57.8 | -24.9 | 35.9 | 100 |
| C12 | 2.403 | 1.158 | 1.050 | 0.120 | 9.3 | 89.7 | 93.7 | 72.8 | -8.5 | 22.3 | 100 |
| C13 | 1.020 | 0.275 | 0.311 | 0.069 | 0.0 | 75.0 | 24.3 | 31.3 | -4.4 | -28.8 | 100 |
| C14 | 2.211 | 1.084 | 1.550 | 0.111 | 0.0 | 89.8 | 52.6 | 62.5 | -13.6 | -18.8 | 100 |
| C15 | 2.299 | 1.021 | 1.068 | 0.115 | 4.7 | 88.7 | 92.8 | 65.7 | -10.4 | 29.2 | 100 |
| C16 | 1.413 | 0.484 | 0.893 | 0.091 | 0.6 | 81.2 | 6.1 | 14.1 | -25.5 | 0 | 0 |
| C17 | 1.393 | 0.409 | 0.433 | 0.071 | 5.8 | 82.6 | 77.5 | 47.1 | -26.0 | 39.3 | 100 |
| C18 | 0.461 | 0.128 | 0.133 | 0.060 | 1.0 | 53.0 | -15.6 | 0.7 | -15.8 | 0 | 0 |
| C19 | 0.846 | 0.241 | 0.212 | 0.066 | 11.8 | 72.5 | 2.1 | -7.2 | -39.7 | 0 | 0 |
| C20 | 2.339 | 0.925 | 1.017 | 0.121 | 0.9 | 87.0 | 87.0 | 44.0 | -36.9 | 49.5 | 100 |
| **Median** | **1.253** | **0.385** | **0.379** | **0.073** | **4.3** | **81.2** | **30.3** | **18.1** | **-19.3** | **41.1** | **100** |
| D1 | 3.182 | 2.484 | 2.561 | 0.877 | 0.0 | 64.7 | 99.2 | 99.5 | 52.6 | -0.3 | 46.9 |
| D2 | 2.476 | 1.184 | 1.311 | 0.308 | 0.0 | 74.0 | 99.1 | 98.8 | 9.6 | 0.3 | 90.3 |
| D3 | 2.527 | 0.988 | 1.189 | 0.221 | 0.0 | 77.6 | 99.0 | 99.2 | -4.0 | -0.2 | 100 |
| D4 | 2.082 | 0.662 | 0.738 | 0.167 | 0.0 | 74.8 | 98.7 | 88.9 | 12.1 | 10.0 | 87.8 |
| D5 | 2.853 | 2.193 | 2.378 | 0.795 | 0.0 | 63.7 | 98.7 | 98.6 | 34.1 | 0.1 | 65.4 |
| D6 | 0.842 | 0.220 | 0.280 | 0.088 | 0.0 | 59.9 | -17.0 | 14.9 | 19.6 | 0 | 0 |
| D7 | 0.848 | 0.214 | 0.237 | 0.089 | 0.0 | 58.3 | -19.4 | 5.1 | -15.4 | 0 | 0 |
| D8 | 1.040 | 0.243 | 0.254 | 0.073 | 0.0 | 70.1 | -8.7 | -1.4 | 5.4 | 0 | 0 |
| D9 | 0.763 | 0.167 | 0.188 | 0.064 | 0.0 | 62.0 | -33.0 | -14.1 | -7.9 | 0 | 0 |
| D10 | 0.769 | 0.050 | 0.241 | 0.087 | 0.0 | 0.0 | -22.6 | -7.1 | -1.8 | 0 | 0 |
| **Median** | **1.561** | **0.453** | **0.509** | **0.128** | **0.0** | **64.2** | **45.0** | **51.9** | **7.5** | **0.1** | **87.8** |
| T1 | 2.465 | 0.505 | 0.523 | 0.201 | 0.0 | 60.2 | 78.9 | 36.8 | -0.6 | 53.4 | 100.0 |
| T2 | 3.238 | 2.846 | 3.340 | 2.511 | 0.0 | 11.8 | 99.5 | 99.8 | 100 | -0.4 | -0.9 |
| T3 | 2.853 | 2.193 | 2.378 | 0.795 | 0.0 | 63.7 | 99.8 | 76.9 | 35.8 | 22.9 | 64.1 |
| T4 | 3.384 | 3.384 | 2.935 | 1.466 | 13.3 | 56.7 | 98.9 | 100 | 99.3 | -1.1 | -0.5 |
| T5 | 3.182 | 2.484 | 2.561 | 0.877 | 0.0 | 64.7 | 100 | 100 | 64.5 | 0.0 | 35.5 |
| T6 | 3.092 | 3.238 | 2.693 | 1.367 | 16.8 | 57.8 | 99.5 | 99.8 | 100 | -0.3 | -0.5 |
| T7 | 3.098 | 1.979 | 2.136 | 0.786 | 0.0 | 60.3 | 99.2 | 99.5 | 84.2 | -0.3 | 15.1 |
| T8 | 2.814 | 1.119 | 1.166 | 0.288 | 0.0 | 74.3 | 100 | 100 | 63.4 | 0.2 | 36.7 |
| T9 | 3.051 | 2.990 | 2.965 | 2.370 | 0.8 | 20.7 | 99.9 | 100 | 98.3 | -0.1 | 1.5 |
| T10 | 2.822 | 1.433 | 1.247 | 0.382 | 13.0 | 73.3 | 100 | 100 | 74.5 | 0.0 | 25.5 |
| **Median** | **3.071** | **2.339** | **2.470** | **0.836** | **0** | **54.4** | **97.6** | **91.3** | **72.0** | **7.4** | **27.7** |

^1^ Reduction of binding: % reduction of IgG binding calculated between RBD-Wuhan and RBD-Delta or RBD-Wuhan and RBD-Omicron (Online repository: Supplemental methods)

^2^ Percent of inhibition of RBD binding to ACE2 by respective sera: +/- 10 % are considered no inhibition, 10-50 % moderate inhibition, >50 % strong inhibition and < -10% shows enhancement of molecular interaction.

^3^ Reduction of respective RBD-ACE2 binding inhibition by the indicated sera: % reduction of inhibition rates calculated between RBD-Wuhan and RBD-Delta or between RBD-Wuhan and RBD-Omicron (Online repository: Supplemental methods)
